# Spelling of Emerging Pathogens

**DOI:** 10.3201/eid1111.050780

**Published:** 2005-11

**Authors:** John E. Moore, B. Cherie Millar

**Affiliations:** * Belfast City Hospital, Belfast, Northern Ireland, United Kingdom

**Keywords:** Q fever, Coxiella, Whipple disease, coccidioidomycosis, letter

**To the Editor:** Language is about comprehension; provided the parties in a discussion can understand each other, variations in pronunciation of individual words may be tolerated or disregarded. In modern English, numerous examples of variant pronunciations exist that cause no problems of comprehension (e.g., either, tomato, laboratory, fertile). These arise from several causes; regional practice is likely the most important factor, but the speaker's education and social background, personal preferences, and even etymologic theories also play a part. It would be futile and, some would feel, undesirable to attempt to impose uniformity by prescribing approved pronunciations if communication is not endangered. Moreover, both language and pronunciation are subject to constant change.

The same is not true regarding the spelling of organisms' names. Although we accept variation in pronunciation, we should not accept variation in the spelling of binomial names. Common spelling variants and the citation frequency (PubMed) of 4 organisms, *Acinetobacter baumannii*, *Coccidioides immitis* (the fungal causal agent of coccidioidomycosis), *Coxiella burnetii* (the causal agent of Q fever), and *Tropheryma whipplei* (the causal agent of Whipple disease), are detailed in the Table. Common spelling mistakes occur with double letters (e.g., nn, ii), as well as complicated strings of consecutive vowels (e.g., *Coccidioides*). However, a defense to such criticism is that various authors have adopted the spelling of a previous taxonomic description that has become outdated, e.g., *C. burneti* (previous) and *C. burnetii* (current). Historic change in the spelling of these names is the primary reason they are published and cited in PubMed with different spellings. However, even disregarding historic taxonomic variants, ≈14.8% of *Tropheryma whipplei*, 14.3% of *Acinetobacter baumannii*, 12.3% of *Coxiella burnetii*, and 1.9% of *Coccidioides* citations are spelled incorrectly in PubMed. These relatively large percentages may mean that relevant literature is overlooked in searches.

**Table:** Common spellings of binomial names of organisms*

Organism name [no. citations in PubMed]*	Spelling variants [no. citations in PubMed]	Date official spelling first described
*Acinetobacter baumannii* [844]	*A. baumanii* [117] *A. baumanni* [18] *A. baumani* [6]	1986†
*Coccidioides*‡ [1,209]	*Coccidiodes* [17] *Coccidoides* [4] *Cocidioides* [2] *Coccidioidis* [1]	1896§
*Coxiella burnetii* [1,531]	*C. burneti* [374] *C. burnetti* [199] *C. burnettii* [16]	1980¶
*Tropheryma whipplei* [52]	*T. whippelii* [118] *T. whippleii* [5] *T. whippeli* [4]	2001#

*Organism name in List of Bacterial Names with Standing in Nomenclature; search conducted June 2005. †Approved name described by Bouvet and Grimont (ref 1). ‡*Coccidioides* is not a bacterium but a fungus; however, this name is described in the Index Fungorum. §First described by Stiles (ref 2). ¶Approved name described by Skerman et al. (ref 3); first described by Derrick (ref 4) as *Rickettsia burneti*, the cause of Q fever. #Approved names described by La Scola et al. (ref 5); 1992, Relman et al. (ref 6) tentatively proposed the name "*T. whippelii*."

Authors should be aware that previous taxonomic spelling of binomial names exist and check their historic evolution in the List of Prokaryotic Names with Standing in Nomenclature (http://www.bacterio.cict.fr). Authors should cite previous spelling when such a change has been recent and they may wish to include previous spellings in literature searches. Additionally, the most current and formally accepted spelling must be used when preparing a manuscript for publication.

The origins of incorrect and variant spellings of binomial names may lie in an array of sources, including original mispronunciation with subsequent incorrect phonetic transcription. Written language is rarely a phonetic transcript of vocal acoustics, however, it interfaces with several factors that prevent us from spelling words the way they sound. Orthography, which promotes the practice of writing words with the proper letters according to standard usage and conventionally correct spelling, is further complicated by the use of Greek or Latin words, each with their own linguistic peculiarities.

Although we may not be able to standardize phonetic pronunciation of binomial names locally, nationally, or internationally, we should be constantly conscious of their spelling. As authors and peer reviewers, we should strive to achieve uniformity in written media to promote enhanced communication with our peers in infectious diseases.
